# The early change of SOFA score as a prognostic marker of 28-day sepsis mortality: analysis through a derivation and a validation cohort

**DOI:** 10.1186/s13054-019-2665-5

**Published:** 2019-11-29

**Authors:** Eleni Karakike, Evdoxia Kyriazopoulou, Iraklis Tsangaris, Christina Routsi, Jean-Louis Vincent, Evangelos J. Giamarellos-Bourboulis

**Affiliations:** 1 4th Department of Internal Medicine, Attikon University Hospital, National and Kapodistrian University of Athens, 1 Rimini Street, 12462 Athens, Greece; 2 2nd Department of Critical Care Medicine, National and Kapodistrian University of Athens, 12462 Athens, Greece; 3 1st Department of Critical Care Medicine, National and Kapodistrian University of Athens, 10676 Athens, Greece; 4 Department of Intensive Care, Erasme Hospital, Université libre de Bruxelles, 1070 Brussels, Belgium

**Keywords:** Sequential Organ Failure Assessment (SOFA), Delta change, Sepsis, Mortality, Trial endpoints

## Abstract

**Background:**

Since the Sepsis-3 criteria, change in Sequential Organ Failure Assessment (SOFA) score has become a key component of sepsis identification. Thus, it could be argued that reversal of this change (Δ_SOFA_) may reflect sepsis response and could be used as measure of efficacy in interventional trials. We aimed to assess the predictive performance of Δ_SOFA_ for 28-day mortality.

**Methods:**

Data from two previously published randomized controlled trials were studied: the first reporting on patients with severe Gram-negative infections as a derivation cohort and the second reporting on patients with ventilator-associated pneumonia as a validation cohort. Only patients with sepsis according to the Sepsis-3 definition were included in this analysis. SOFA scores were calculated on days 1, 2, 3, 5, 7, 14, and 28.

**Results:**

We included 448 patients within the derivation cohort and 199 within the validation cohort. Mean SOFA scores on day 1 were 6.06 ± 4.07 and 7.84 ± 3.39, and 28 day mortality 22.8% and 29.6%, respectively. In the derivation cohort, the earliest time point where Δ_SOFA_ score predicted mortality was day 7 (AUROC (95% CI) 0.84 (0.80–0.89); *p* < 0.001). The best tradeoff for prediction was found with 25% changes (78% sensitivity, 80% specificity); less than 25% decrease of admission SOFA was associated with increased mortality (odds ratio for death 14.87). This finding was confirmed in the validation cohort.

**Conclusions:**

Δ_SOFA_ on day 7 is a useful early prognostic marker of 28-day mortality and could serve as an endpoint in future sepsis trials alongside mortality.

**Trial registration:**

ClinicalTrials.gov numbers NCT01223690 and NCT00297674

## Background

In the light of numerous inconclusive interventional clinical trials in sepsis during the past two decades, the framework of those trials is to be revised [1–4]. All-cause mortality after 28 days has traditionally been the primary endpoint in these trials. However, with recent improvements in standard-of-care therapy, 28-day mortality is strongly dependent from other variables such as comorbid conditions and the adverse events of multiple interventions [5]. As such, it is reasonable that alternative endpoints need to be developed for sepsis. These endpoints need to provide earlier and accurate evaluation of the treatment effect under study.

Since sepsis is triggered by an infection, the endpoint of sepsis trials may be influenced by the attitude of regulatory bodies to focus new registration trials of antimicrobial agents towards early efficacy. The main example towards this end is the joint initiative between the Food and Drug Administration (FDA) with the Biomarkers Consortium of the Foundation for the National Institutes of Health (FNIH) on the update of primary endpoint definitions for non-inferiority trials for the management of infectious diseases. More precisely, the former test-of-cure visit usually taking place 7–14 days after end of treatment was replaced by the early response 48–72 h after start of treatment for acute bacterial skin and soft structure infections [6] and 3–5 days after start of treatment for community-acquired pneumonia [7], while efforts are being made to expand this concept to hospital-acquired and ventilator-associated pneumonia [8, 9]. However, in order to develop a similar early endpoint for sepsis, it is mandatory that this endpoint is a predictor of 28-day mortality, i.e., the salient sequelum of sepsis and eventually of 90-day mortality that has recently emerged as a relevant clinical endpoint [10]. With the Sepsis-3 classification criteria, the Sequential Organ Failure Assessment (SOFA) score is used as a measure of sepsis-associated organ dysfunction. As a consequence, it is reasonable to define the earliest time point during the course of the disease where a clinical meaningful change of the baseline SOFA score is achieved.

The present study tries to define (a) the earliest time point during the course of sepsis where SOFA score changes can predict 28-mortality and (b) the cutoff change of baseline SOFA score that may be considered an early sign of sepsis resolution. The association of SOFA score changes with 90-day mortality is also assessed. In order to achieve so, we used two independent prospective cohorts of patients: the first as a derivation cohort and the second as a validation cohort.

## Patients and methods

### Study populations

We retrospectively analyzed clinical data from a cohort of patients with sepsis, according to the 1991 sepsis definitions (derivation cohort) [11]; a second independent cohort using the 1991 sepsis definitions served as validation dataset for the primary hypothesis. Both cohorts were part of previously published multicenter randomized controlled trials comparing clarithromycin to placebo as adjunctive immunomodulatory treatment in sepsis [12, 13].

The derivation cohort included patients with Gram-negative sepsis, enrolled in a prospective double-blind, placebo-controlled randomized clinical trial (RCT) studying the efficacy of intravenous clarithromycin in 28-day mortality. Patients were recruited from July 2007 to August 2011 in six departments (two intensive care units—ICUs, three medical wards, and one surgical ward) in five tertiary teaching hospitals in Greece. Patients were suffering from acute pyelonephritis or intra-abdominal infections or primary Gram-negative bacteremia [12] (ClinicalTrials.gov NCT01223690). Since the 28-day mortality of patients allocated to the placebo arm and of patients allocated to the clarithromycin arm did not differ, both arms were analyzed together for the purpose of this study.

The validation cohort consisted of patients with ventilator-associated pneumonia (VAP), enrolled in an RCT in two ICUs (one patient enrolled in one medical ward has not been included in the present study) in two tertiary teaching hospitals in Greece, from June 2004 to November 2005 (ClinicalTrials.gov NCT00297674) [13]. Since the 28-day mortality of patients allocated to the placebo arm and of patients allocated to the clarithromycin arm did not differ, both arms were analyzed together.

All medical and nursing charts of the derivation cohort were retrospectively reviewed, and components of SOFA score for each system (respiratory, coagulation, liver, cardiovascular, central nervous, and renal) were collected. Serial SOFA scores were calculated initially on day 1 (initial SOFA) and on days 2, 3, 5, 7, 14, and 28 after enrollment in the study.

For the purposes of this study, patients of each cohort who were meeting the Sepsis-3 criteria were identified; only those participated in this analysis. For the calculation of serial SOFA scores, when the Glasgow Come Scale (GCS) was not evaluable due to sedation for mechanical ventilation, the GCS immediately before mechanical ventilation was used. Patients discharged from hospital or deceased before day 28 were censored to the last known SOFA score. Delta SOFA (Δ_SOFA_) for any follow-up day was provided by the formula: (SOFA score of the follow-up day − initial SOFA score) × 100/day 1 SOFA, and it was expressed as percentage.

The outcome measure in both cohorts was the earliest time point where the change of SOFA score was associated with 28-day mortality. The association of this change with 90-day mortality was a secondary endpoint.

### Statistical analysis

Categorical values were presented as percentages, and continuous variables with normal distribution as mean and standard deviation (± SD). Categorical variables were compared using the two-sided Fisher exact test, whereas quantitative variables were assessed using Student’s *t* test or the non-parametric Mann-Whitney test, as appropriate. The predictive capacity of different follow-up day Δ_SOFA_ for mortality was evaluated with the area under the respective receiver operator characteristics (AUROC) curves and 95% confidence intervals (CIs). The optimal cutoff value for prediction of 28-day mortality was calculated using Youden’s index. The Δ_SOFA_ was expressed by medians and 95% CIs; comparisons between survivors and non-survivors were done by the Mann-Whitney *U* test. Breslow-Day's test was used to compare the performance of this cutoff value between the derivation and validation cohorts. A *p* value lower than 0.05 was considered statistically significant. All *p* values were two-sided. Statistical analyses were performed using SPSS version 25.0 software.

## Results

**Fig. 1:**
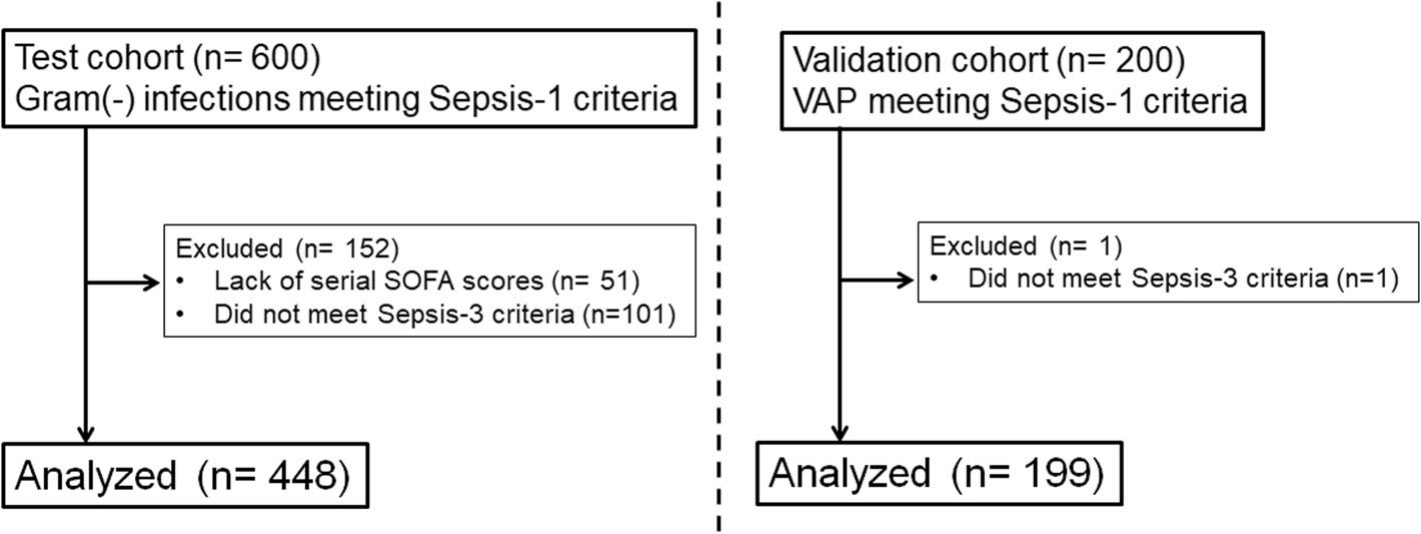
Flow chart. VAP, ventilator-associated pneumonia; SIRS, systemic inflammatory response syndrome; SOFA, sequential Organ Failure Assessment

The study flow charts for both cohorts are shown in Fig. 1. A total of 448 of patients of the derivation cohort and 199 patients of the validation cohort could be classified as sepsis according to the Sepsis-3 criteria and were included in the analysis. Demographic baseline data of the two cohorts differed significantly (Table 1).

**Table 1:** Comparative baseline demographics of the two cohorts

	Derivation cohort (*n* = 448)	Validation cohort (*n* = 199)	*p* value
Male gender, *n* (%)	213 (47.5)	147 (73.9)	< 0.001
Age (years, mean ± SD)	71.7 ± 16.6	58.4 ± 19.1	< 0.001
SOFA score (mean ± SD)	6.1 ± 4.1	7.8 ± 3.4	< 0.001
APACHE II score (mean ± SD)	15.1 ± 7.4	17.1 ± 5.7	0.001
CCI (mean ± SD)	4.1 ± 2.5	2.6 ± 1.7	< 0.001
PaO2/FiO2 ratio (mean ± SD)	298.8 ± 112.6	218.5 ± 98.0	< 0.001
Mechanical ventilation, *n* (%)	90 (20.1)	199 (100)	< 0.001
Characteristics of MV population			
Tidal volume (ml/kg, mean ± SD)	6.6 ± 0.9	6.5 ± 0.9	0.179
PEEP level (mmHg, mean ± SD)	5.7 ± 0.9	6 ± 0.9	0.011
PaO2/FiO2 ratio (mean ± SD)	252.7 ± 113.7	218.5 ± 98.0	0.020
Duration of MV (days, mean ± SD)	14.5 ± 13.8	14.7 ± 10.4	0.346
Underlying infection, *n* (%)			
Acute pyelonephritis	207 (46.2)	0 (0)	NA
Acute intra-abdominal infection	162 (36.2)	0 (0.0)	NA
Primary Gram-negative bacteremia	71 (15.8)	0 (0.0)	NA
Secondary Gram-negative bacteremia (other than urinary or intra-abdominal)	8 (1.8)	0 (0.0)	0.107
Ventilator-associated pneumonia	0 (0)	199 (100.0)	NA
Early (< 7 days of MV)		84 (42.2)	
Late (> 7 days of MV)		115 (57.8)	
Septic shock, *n* (%)	88 (19.6)	85 (42.7)	< 0.001
ARDS, *n* (%)	136 (30.4)	150 (75.4)	< 0.001
ICU admission, *n* (%)	90 (20.1)	198 (99.5)	< 0.001
ICU LOS (days, mean ± SD)	45.3 ± 94.3	36.9 ± 34.4	0.317
Hospital LOS (days, mean ± SD)	20.0 ± 47.6	51.7 ± 47.5	< 0.001
For ICU-admitted population	49.6 ± 98.9	51.7 ± 47.5	0.006
For non-ICU-admitted population	12.6 1 ± 0.9	NA	
ICU mortality, *n* (%)	49 (54.4)	89 (44.9)	0.162
Hospital mortality, *n* (%)	123 (27.5)	110 (55.3)	< 0.001
28-day mortality, *n* (%)	102 (22.8)	59 (29.6)	0.075
90-day mortality, *n* (%)	118 (26.3)	153 (76.9)	< 0.001

*Abbreviations*: *ARDS*: Acute Respiratory Distress Syndrome, *SD* standard deviation, *SOFA* Sequential Organ Failure Assessment, *APACHE* Acute Physiology and Chronic Health Evaluation, *CCI* Charlson’s comorbidity index, *ICU*: Intensive Care Unit, *LOS*: Length of Stay, *MV: mechanical ventilation, NA* not applicable

### Primary endpoint

**Fig. 2:**
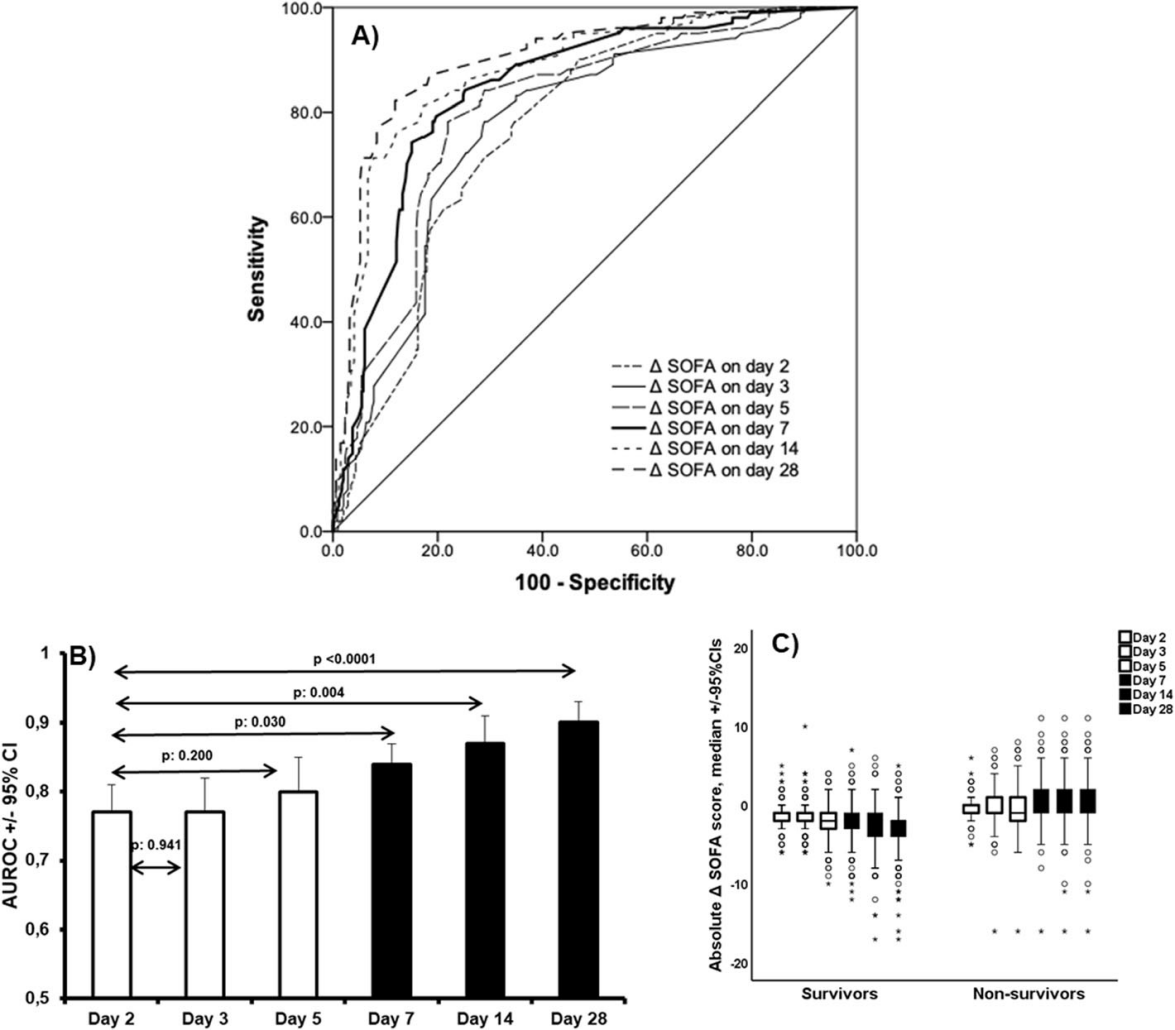
Δ_SOFA_ on follow-up days as predictor of 28-day mortality in the derivation cohort. **a** Receiver operating characteristic (ROC) curves for the association of change from initial SOFA (Δ_SOFA_) with 28-day mortality. **b** Comparisons of AUROCs of Δ_SOFA_ of follow-up days to Δ_SOFA_ of day 2. *p* values of the indicated comparisons are provided. **c** Median Δ_SOFA_ scores on follow-up days in survivors and non-survivors. Statistically significant differences at the level of *p* < 0.0001 were found between survivors and non-survivors at all studied time points. AUROC, area under the ROC; CI, confidence interval

The ROC curves of the performance of Δ_SOFA_ of follow-up days for the prediction of 28-day mortality in the derivation cohort are shown in Fig. 2a. When the AUROCs of Δ_SOFA_ of follow-up days were compared, it was found that the earliest time point when the achieved AUROC was greater than previous days was on day 7 (Fig. 2b). When the absolute Δ_SOFA_ scores were compared over time between survivors and non-survivors, despite the significantly greater decreases in survivors from non-survivors found by non-parametric statistics at all time points, a great overlap of values was shown (Fig. 2c). This led us to consider the percentage change of baseline SOFA as a more appropriate expression of the sepsis course than the absolute Δ_SOFA_. To this end, our analysis focused on the development of a specific value of Δ_SOFA_ of day 7 as an early predictor of 28-day mortality. The analysis using the Youden index showed that a 25% cutoff value could discriminate non-survivors from survivors with sensitivity 78.4% (95% CI 69.0–85.7%), specificity 80.3% (95% CI 75.7–84.3%), positive predictive value 54.1% (95% CI 45.7–62.2%), and negative predictive value 92.7% (95% CI 89.0–95.2%).

Overall, in the derivation cohort, 148 (33%) patients had less than 25% decrease of SOFA score on day 7 and 300 (77%) patients had at least 25% decrease of initial SOFA score on day 7. Mortality after 28 days was 54.1% and 7.3%, respectively (*p* = 1.8361 × 10^−27^). The OR for death after 28 days with a decrease of initial SOFA on day 7 less than 25% was 14.87 (95% CI 8.65–25.54). Similarly, the OR for death in the validation cohort was 6.95 (95% CI 2.05–23.55) (*p* value of the Breslow-Day test of homogeneity 0.250) (Table 2).

**Table 2:** Comparative prognostic performance for 28-day mortality of the less than 25% SOFA decrease cutoff on day 7 Δ_SOFA_ between the derivation and the validation cohorts

	Derivation cohort (95% CI)	Validation cohort (95% CI)	*p* value
Sensitivity	78.4% (69.0–85.7)	93.2% (84.7–98.7)	0.06
Specificity	80.3% (75.7–84.3)	37.9% (20.1–35.4)	2.87 × 10^−28^
PPV	54.1% (45.7–62.2)	38.7% (27.7–43.1)	0.01
NPV	92.7% (89.0–95.2)	93.0% (79.0–98.1)	1.00

*Abbreviations*: *CI* confidence interval, *NPV* negative predictive value, *PPV* positive predictive value

### Secondary endpoint

After ROC analysis, the day 7 Δ_SOFA_ in the derivation cohort yielded an AUROC of 0.847 (0.807–0.886; *p* = 5.11 × 10^−29^) for predicting 90-day mortality. When applying the cutoff of less than 25% decrease, this was associated with an OR of 13.20 for death after 90 days (95% CI 8.01–21.76; *p* = 4.78 × 10^−28^). Table 3 describes the performance characteristics of the cutoff in predicting 90-day mortality in both cohorts.

**Table 3:** Comparative prognostic performance for 90-day mortality of the less than 25% SOFA decrease cutoff on day 7 Δ_SOFA_ between the derivation and validation cohorts

	Derivation cohort (95% CI)	Validation cohort (95% CI)	*p* value
Sensitivity	74.6% (69.5–83.4)	77.1% (69.5–83.4)	0.63
Specificity	81.8% (77.1–85.7)	47.8% (33.1–62.9)	2.00 × 10^−5^
PPV	59.5% (51.1–67.4)	83.1% (75.7–88.7)	9.00 × 10^−5^
NPV	90.0% (85.9–93.1)	38.6% (26.3–52.4)	1.54 × 10^−27^

*Abbreviations*: *CI* confidence interval, *NPV* negative predictive value, *PPV* positive predictive value

### Post hoc analysis

Although the validation cohort involved 199 with VAP all of whom were under mechanical ventilation, the derivation cohort comprised both mechanically (*n* = 71) and non-mechanically ventilated patients (*n* = 377) on study enrollment. The 28-day mortality among mechanically ventilated patients with at least 25% decrease of initial SOFA score and among mechanically ventilated patients with less than 25% decrease of initial SOFA score was 11.5% and 37.8%, respectively (*p* = 0.027). The respective 28-day mortality among the non-mechanically ventilated patients was 7.0% and 60.0%, respectively (*p* = 1.1 × 10^−26^).

Due to the significant baseline differences between the derivation and validation cohorts and in order to assess the robustness of the above findings, a post hoc analysis has been performed, by merging both initial cohorts and randomly splitting them into cohort A and cohort B. It needs to be outlined that patients of both original cohorts were recruited before 2012 (the first in the years 2004–2005 and the second in the years 2007–2011). The standard-of-care for patients remained approximately the same between these two periods since the Surviving Sepsis Campaign guidelines remained largely unchanged between 2004 and 2008 as also where national recommendations for antimicrobial use. Baseline characteristics of the new cohorts shown in Additional file 1: Table S1 did not differ. The 25% change of initial SOFA score worked equally well for the prediction of both 28-day and 90-day mortality in both cohorts A and B (Table 4 and Additional file 2: Table S2, respectively).

**Table 4:** Prognostic performance for 28-day mortality of the 25% SOFA decrease cutoff on day 7 Δ_SOFA_ using post-hoc derivation and validation cohorts

	Cohort A		Total	Cohort B		Total
	Non-survivors (*n*)	Survivors (*n*)		Non-survivors (*n*)	Survivors (*n*)	
≥ 25% SOFA decrease	74 Sens: 86.0% PPV: 51.7%	69	143	61 Sens: 91.3% PPV: 41.5%	86	147
< 25% SOFA decrease	12	169 Spec:71.0% NPV: 93.4%	181	14	162 Spec: 65.3% NPV: 92.0%	176
	86	238	324	75	248	323

*Abbreviations*: *NPV* negative predictive value, *PPV* positive predictive value, *Sens* sensitivity, *Spec* specificity

Another concern was that some investigators handle SOFA score for deceased patients as the last observation carried forward, while others set the score to 24 in case of death. Using the second approach in the derivation cohort, it was found that 28-day mortality among 295 patients with at least 25% decrease of initial SOFA score was 6.1%; this was 56.2% among 153 patients with less than 25% decrease of the initial SOFA score.

## Discussion

To the best of our knowledge, this is the first study to report a specific cutoff of 25% decrease of SOFA score as the earliest significant surrogate of 28-day mortality using a derivation and a validation cohort. The cutoff remained robust in all subsequent analyses and subgroup evaluations, despite the fact that the used cohorts differed considerably in baseline characteristics, indicating that the elaborated endpoint may be generalizable.

Previous studies have shown that serial SOFA measurements are predictors of mortality on both days 3 and 5 of follow-up [14, 15]. A cohort study of 20,007 critically ill patients in Canada reported that the slope of the SOFA score between days 1 and 7 was higher and better associated with final outcome (both ICU and hospital mortality) than was the average rate of change at later time points (between days 8 and 14) [16]. According to the authors, any increase between days 1 and 5 (defined as early change) was significantly associated with hospital and ICU mortality.

Recently, in a meta-regression analysis from 87 RCTs on septic patients using different SOFA derivatives as primary or secondary endpoints, the authors have shown that Δ_SOFA_ (when defined as a fixed day minus initial day SOFA) explained 32% of treatment effect on mortality, suggesting that Δ_SOFA_ is both responsive and consistent in detecting differences of treatment effects on mortality and could replace mortality as a surrogate endpoint in clinical trials [17]. The validity of change of SOFA on day 7 as an early predictor of 28-day mortality was analyzed in a large post-marketing survey among patients with sepsis and disseminated intravascular coagulation, who were propensity-matched to receive either antithrombin III alone or combination therapy with thrombomodulin. Although no difference was found between the two groups, when they were analyzed together, it was found that these changes between day 1 and day 7 provided AUROC 0.81 for 28-day mortality [18]. In a cohort of severe sepsis and septic shock, day 3 Δ_SOFA_ displayed AUROC 0.68 (95% CI 056–0.79) whereas 50% SOFA decrease was associated with 61.3% sensitivity and 85.9% negative predictive value for ICU mortality prediction [19].

Another suggested endpoint based on SOFA score is the mean total SOFA score. This is the sum of the follow-up day SOFA scores divided by the number of days of ICU stay. In an historical cohort of 352 patients with mean length of stay (LOS) of 6.5 days, the mean total SOFA correlated well with mortality (OR 3.06, 95% CI 2.36 to 3.97) [15]. In a study evaluating levosimendan compared to placebo in patients with septic shock (the LeoPARDS RCT), the primary endpoint was powered to detect an absolute difference in the mean SOFA score (calculated up to a maximum of 28 ICU days) of at least 0.5 between the two arms [20]. The MaxSep RCT, comparing meropenem alone or in combination with moxifloxacin, in patients with severe sepsis, aimed to demonstrate a minimum of 1.1 point difference in mean SOFA scores between the two arms (calculated for a maximum ICU stay of 14 days) [21]. Both studies failed to demonstrate the expected difference, despite adequately large sample sizes (more than 500 patients per study), possibly due to the cutoffs used.

In the light of the existing publications, it is obvious that the suggested cutoff of at least 25% decrease of SOFA score on day 7 may neither replace mortality as an endpoint of clinical trials nor be considered a surrogate for sepsis resolution. However, there is no doubt that it may be considered as an early marker of improvement of the sepsis process so as to be encountered alongside mortality.

One major limitation of our study is the retrospective analysis of the data. However, due to the fact that all included patients were part of a prospective follow-up protocol during the initial randomized clinical trials, all required data were systematically collected up to day 28 limiting the bias that may come from this approach.

## Conclusions

Overtime changes in Sequential Organ Failure Assessment score (Δ_SOFA_) offer a more direct, scalar measurement of treatment effect of sepsis compared to traditional mortality endpoints. Any less than 25% Δ_SOFA_ on day 7 may identify high mortality-risk patients showing that Δ_SOFA_ changes may be incorporated alongside mortality in future clinical trials.

## Supplementary information

**Additional file 1: Table S1.** Comparative demographics of the two novel cohorts.

**Additional file 2: Table S2.** Prognostic performance for 90-day mortality of the 25% SOFA decrease cutoff on day 7 Δ_SOFA_ between the derivation and validation cohorts.

## Data Availability

The datasets used and/or analyzed during the current study are available from the corresponding author upon reasonable request.

## Abbreviations

APACHE: Acute Physiology and Chronic Health Evaluation
ARDS: acute respiratory distress syndrome
AUROC: Area under the receiver operating characteristics curve
CCI: Charlson’s comorbidity index
CI: Confidence intervals
Δ_SOFA_: Delta (change in) SOFA
FDA: Food and Drug Administration
FNIH: Foundation for the National Institutes of Health
GCS: Glasgow Coma Scale
HIV: Human immunodeficiency virus
ICU: Intensive care unit
LOS: Length of stay
MV: mechanical ventilation
NPV: Negative predictive value
OR: Odds ratio
PPV: Positive predictive value
RCT: Randomized clinical trial
ROC: Receiver operating characteristics
SD: Standard deviation
SIRS: Systemic inflammatory response syndrome
SOFA: Sequential Organ Failure Assessment
VAP: Ventilator-associated pneumonia
