# Supplementary material for: The early change of SOFA score as a prognostic marker of 28-day sepsis mortality: analysis through a derivation and a validation cohort
Source: Crit Care. 2019 Nov 29;23:387. doi: 10.1186/s13054-019-2665-5 (PMC6884794; doi:10.1186/s13054-019-2665-5)
Supplement: Supplementary file 1 — Additional file 1: Table S1. Comparative demographics of the two novel cohorts. [file 13054_2019_2665_MOESM1_ESM.docx]

**Additional File 1: Table S1** Comparative demographics of the two novel cohorts

|  | **New cohort A (n=324)** | **New cohort B (n=323)** | **p-value** |
| --- | --- | --- | --- |
| Male gender, n (%) | 189 (58.3) | 170 (52.6) | 0.155 |
| Age (years, mean ±SD) | 67.3 ± 18.1 | 69.1 ± 17.7 | 0.131 |
| Initial SOFA score (mean ± SD) | 6.7 ± 4.0 | 6.6 ± 3.9 | 0.989 |
| APACHE II score (mean ± SD) | 15.7 ± 7.0 | 15.8 ± 6.9 | 0.819 |
| CCI (mean ± SD) | 3.6 ± 2.4 | 3.7 ± 2.4 | 0.662 |
| PaO2/ FiO2 ratio (mean ± SD) | 262.7 ± 115.3 | 269.0 ± 112.5 | 0.466 |
| Mechanical ventilation, n (%) | 153 (47.2) | 136 (42.1) | 0.206 |
| Characteristics of MV population |  |  |  |
| Tidal volume (ml/kg, mean ± SD) | 6.5 ± 0.9 | 6.6 ± 0.9 | 0.255 |
| PEEP level (mmHg, mean ± SD) | 6.0 ± 1.0 | 5.9 ± 0.9 | 0.443 |
| PaO2/FiO2 ratio (mean ± SD) | 225.2 ± 103.6 | 233.6 ± 105.0 | 0.540 |
| Duration of MV (days, mean ± SD) | 14.4 ± 10.7 | 15.0 ± 11.8 | 0.830 |
| Underlying infection, n (%) |  |  | 0.176 |
| Acute pyelonephritis | 95 (29.3) | 112 (34.7) | 0.144 |
| Acute intra-abdominal infection | 76 (23.5) | 86 (26.6) | 0.352 |
| Primary Gram-negative bacteremia | 35 (10.8) | 36 (11.1) | 0.889 |
| Secondary Gram-negative bacteremia | 4 (1.2) | 2 (0.7) | 0.686 |
| Ventilator-associated pneumonia | 114 (35.2) | 87 (26.9) | 0.023 |
| Early (<7 days of MV) | 44 (38.6) | 41 (47.1) | 0.225 |
| Late (>7 days of MV) | 70 (61.4) | 46 (52.9) |  |
| Septic shock, n (%) | 90 (27.8) | 74 (22.9) | 0.175 |
| ARDS, n (%) | 144 (44.4) | 142 (44.0) | 0.937 |
| ICU admission, (n%) | 152 (46.9) | 136 (42.1) | 0.236 |
| ICU LOS (days, mean ± SD) | 34.1 ± 33.4 | 45.6 ± 79.2 | 0.195 |
| Hospital LOS (days, mean ± SD) | 26.3 ± 32.9 | 33.2 ± 62.0 | 0.360 |
| for ICU admitted population | 42.1 ± 41.4 | 61.2 ± 87.4 | 0.026 |
| for non-ICU admitted population | 12.3 ± 10.7 | 12.8 ± 11.2 | 0.392 |
| ICU mortality, n (%) | 78 (51.3) | 60 (44.1) | 0.239 |
| Hospital mortality, n (%) | 121 (37.3) | 112 (34.7) | 0.513 |

| 28-day mortality, n (%) | 86 (26.5) | 75 (23.2) | 0.363 |
| --- | --- | --- | --- |

| 90-day mortality, n (%) | 144 (44.4) | 127 (39.3) | 0.203 |
| --- | --- | --- | --- |

Abbreviations APACHE: acute physiology and chronic health valuation; ARDS: acute respiratory distress syndrome; CCI: Charlson’s comorbidity index; ICU: intensive care unit; LOS: length of stay; MV: mechanical ventilation; SD: standard deviation; SOFA: sequential organ failure assessment;
