# Supplementary material for: The early change of SOFA score as a prognostic marker of 28-day sepsis mortality: analysis through a derivation and a validation cohort
Source: Crit Care. 2019 Nov 29;23:387. doi: 10.1186/s13054-019-2665-5 (PMC6884794; doi:10.1186/s13054-019-2665-5)
Supplement: Supplementary file 2 — Additional file 2: Table S2. Prognostic performance for 90-day mortality of the 25% SOFA decrease cutoff on day 7 ΔSOFA between the derivation and validation cohorts. [file 13054_2019_2665_MOESM2_ESM.docx]

**Additional file 2: Table S2 Prognostic performance for 90-day mortality of the 25% SOFA decrease cutoff on day 7 Δ_SOFA_ between the derivation and validation cohorts**

|  | Cohort A | | Total | Cohort B | | Total |
| --- | --- | --- | --- | --- | --- | --- |
|  | Non-survivors (n) | Survivors (n) |  | Non-survivors (n) | Survivors (n) |  |
| ≥25% SOFA decrease | 110  Sens: 76.4%  PPV: 76.9% | 33 | 143 | 96  Sens: 76.6%%  PPV: 65.3% | 51 | 147 |
| <25% SOFA decrease | 34 | 147  Spec: 81.7%  NPV: 81.2% | 181 | 31 | 145  Spec: 74.0%  NPV: 82.4% | 176 |
|  | 144 | 180 | 324 | 127 | 248 | 323 |

Abbreviations: NPV: Negative predictive value; PPV: positive predictive value; Sens: sensitivity; Spec: specificity
